# Molecular Serotype-Specific Identification of Non-type b *Haemophilus influenzae* by Loop-Mediated Isothermal Amplification

**DOI:** 10.3389/fmicb.2017.01877

**Published:** 2017-10-04

**Authors:** Chika Takano, Mitsuko Seki, Dong Wook Kim, Paul E. Kilgore, Kazumasa Fuwa, Koji Takahashi, Toshiaki Inazaki, Satoshi Hayakawa

**Affiliations:** ^1^Division of Microbiology, Department of Pathology and Microbiology, Nihon University School of Medicine, Tokyo, Japan; ^2^Department of Pharmacy, College of Pharmacy, Hanyang University, Ansan, South Korea; ^3^Institute of Pharmacological Research, Hanyang University, Ansan, South Korea; ^4^Department of Pharmacy Practice, Eugene Applebaum College of Pharmacy and Health Sciences, Wayne State University, Detroit, MI, United States; ^5^Medical Devices Solutions Vehicle, Kaneka Corporation, Hyogo, Japan; ^6^Nihon University School of Medicine, Tokyo, Japan

**Keywords:** loop-mediated isothermal amplification, *Haemophilus influenzae*, serotype identification, meningitis, cerebrospinal fluid

## Abstract

Over the past four decades, the incidence of meningitis caused by *Haemophilus influenzae* in children has decreased due to widespread vaccination against *H. influenzae* type b (Hib). The incidence of invasive diseases due to *H. influenzae* types not included in the vaccines, however, has increased. At present, there are a limited number of diagnostics available to detect non-type b *H. influenzae*. To address this issue, we developed a rapid, simple, and cost-effective method for detecting serotypes of *H. influenzae.* We designed LAMP primer sets based on published sequences for *H. influenzae* capsular types a, c, d, e, and f. The assay was evaluated to determine test reactivity, specificity, and sensitivity. To support its use in patients with suspected meningitis, we evaluated the detection limit of the non-Hib serotype specific LAMP assay using bacterial genomic DNA-spiked cerebrospinal fluid (CSF) specimens. The reactivity and specificity of the LAMP assays were confirmed using six serotypes and non-typeable *H. influenzae* strains, plus eight strains of other *Haemophilus* species and non-*Haemophilus* genera. The detection limits of the LAMP assay for capsular types a, c, d, e, and f were 10^2^, 10^2^, 10^2^, 10^3^, and 10 copies per reaction, while those of the PCR assay were 10^4^, 10^4^, 10^3^, 10^3^, and 10^4^ genome copies per reaction, respectively. Using DNA-spiked CSF specimens, the detection limit of the LAMP assay was equivalent to that using purified DNA as the template. However, the detection limit of the PCR was reduced from 10^3^ to 10^4^ genome copies per reaction for serotype d and from 10^3^ to 10^5^ genome copies per reaction for serotype e. To the best of our knowledge, this is the first report of a serotype-specific identification assay for *H. influenzae* using the LAMP method. Our results suggest the potential of LAMP methods for patients with suspected meningitis in resource-limited laboratories or public health surveillance systems.

## Introduction

*Haemophilus influenzae* causes meningitis, epiglottitis, bacteremia, and pneumonia predominantly in infants and young children (Peltola, 2000). Over the past two decades, the introduction of Hib conjugate vaccines into routine immunization schedules has dramatically reduced the incidence of Hib-associated disease in many countries (Barbour, 1996). The incidence of invasive diseases due to *H. influenzae* serotypes not included in the vaccines (non-Hib strains), however, has increased (Bajanca et al., 2004; Rahman et al., 2008; Rubach et al., 2011).

Close monitoring of invasive *H. influenzae* disease continues to be important in order to understand effectiveness of Hib vaccines and detect emergence of invasive *H. influenzae* disease due to non-Hib strains (Ulanova and Tsang, 2014; Desai et al., 2015). To assess *H. influenzae* serotypes carriage, there is a need for laboratory facilities that can reliably cultivate *H. influenzae* and identify the capsular polysaccharide type using immunological techniques. Such facilities are found in well-equipped clinical microbiology laboratories, but serotyping of the capsular antigen can produce inconsistent results (LaClaire et al., 2003; Kim et al., 2011). Accurate *H. influenzae* serotyping remains a challenge due to the limited availability of serotyping methods of the capsular antigen and injudicious use of antimicrobial agents. For this reason, a reliable, non-culture based diagnostic test based on nucleic acid amplification methods has the potential to offer an important option for detection of Hib and non-Hib strains.

A PCR assay for *H. influenzae* serotyping (Falla et al., 1994) has been established, however, PCR-based assays are relatively expensive and complex to perform in resource-limited laboratory settings. An alternative nucleic acid detection method known as loop-mediated isothermal amplification (LAMP) utilizes a unique priming mechanism that yields specific DNA products in a shorter period of time than PCR (Notomi et al., 2000). A LAMP method for detecting Hib has been established and its clinical usefulness confirmed using clinical cerebrospinal fluid specimens (CSF) (Kim et al., 2011).

The non-Hib serotyping LAMP method has the potential to be more reliable and easier to perform than bacterial culture, antigen detection, and PCR-based assays. However, to date, a non-Hib-specific LAMP assay has not been reported. In this study, we report the establishment of a novel non-Hib serotyping LAMP assay, and compare its performance to that of conventional PCR. We also confirmed the performance of the non-Hib serotyping LAMP assay using DNA-spiked clinical CSF specimens from patients with suspected meningitis (Kennedy et al., 2007).

## Materials and Methods

### Bacterial Strains

Forty-two strains of *H. influenzae* (including serotypes a to f, non-typeable and biotype aegyptius) plus eight strains of other *Haemophilus* species and non-*Haemophilus* genera were evaluated. Additional *Haemophilus* species were *H. parainfluenzae* (HK79), *H. parahaemolyticus* (GTC1529), and *H. haemolyticus* (HK680), and non-*Haemophilus* genera were *Streptococcus mitis* (ATCC9811), *S. gordonii* (ATCC12396), *S. pneumoniae* (R6), *Escherichia coli* (DH5α), and *Neisseria meningitidis* (HY0001). In this study, 8 standard and 34 reference *H. influenzae* strains (9 Hib, and 25 non-Hib serotypes, as well as 8 non-typeable strains including 1 biotype aegyptius) were evaluated (**Table 1**). The eight standard *H. influenzae* strains were IID983 (serotype a), IID984 (serotype b), IID985 (serotype c), IID986 (serotype d), IID987 (serotype e), IID 988 (serotype f), IID989 (non-typeable) and IID993 (non-typeable, biotype aegyptius). The 34 reference strains included 5 serotype a (HK390, HK643, HK645, HK648, and HK649), 8 serotype b (HK176, HK177, HK179, HK180, HK195, HK196, HK827, and HK838), 4 serotype c (HK342, HK396, HK635, and HK658), 1 serotype d (HK343), 3 serotype e (HK399, HK636, and HK641), 7 serotype f (HK638, HK2055, HK2056, HK2062, HK2063, HK2065, and HK2109), and 6 non-typeable (HK856, HK2112, HK2115, HK2117, HK2119, and HK2121) strains. Capsule production by the 34 reference strains was previously confirmed by agglutination using burro antisera against serotypes a to f, provided by Dr. Rachel Schneerson (NIH, Bethesda, MD, United States) (**Table 1**).

**Table 1:** Reactivity and specificity of the non-Hib serotyping LAMP assay.

Organism	Number of strains	Origin of isolate^a^	Capsule type^b^	Non-Hib serotyping LAMP assay				
				a	c	d	e	f
Standard *H. influenzae* strains (*n* = 8)^d^	1	—	a	+^c^	-	-	-	-
	1	—	b	-	-	-	-	-
	1	—	c	-	+	-	-	-
	1	—	d	-	-	+	-	-
	1	—	e	-	-	-	+	-
	1	—	f	-	-	-	-	+
	2	—	nt	-	-	-	-	-
Reference *H. influenzae* strains (*n* = 34)^e^	5	RT	a	+	-	-	-	-
	8	CSF	b	-	-	-	-	-
	4	CSF	c	-	+	-	-	-
	1	—	d	-	-	+	-	-
	3	—	e	-	-	-	+	-
	1	CSF	f	-	-	-	-	+
	6	RT	f	-	-	-	-	+
	6	RT	nt	-	-	-	-	-

^a^*RT, respiratory tract specimen; CSF, cerebrospinal fluid. ^b^Capsule types were confirmed by *H. influenzae* serotyping PCR (Falla et al., 1994). nt, non-typeable. ^c^+, positive, amplification after a 35min incubation; -, negative, no amplification after a 60 min incubation. ^d^Source: Institute of Medical Science, University of Tokyo, Tokyo, Japan. ^e^Provided by Prof. Emeritus Mogens Kilian, Biomedicine, Aarhus University, Aarhus, Denmark*.

### Preparation of Chromosomal DNA

Genomic DNA was purified from the 50 strains above using a QIAamp DNA Mini Kit (QIAGEN, Valencia, CA, United States), in accordance with the manufacturer’s protocol. For the detection limit study, genomic DNA from five non-Hib serotypes (IID983, IID985, IID986, IID987, and IID 988) was obtained as described above and the concentration was determined using a NanoDrop 1000 instrument (Thermo Fisher Scientific Inc., Waltham, MA, United States). The number of genome copies in the LAMP mixture was calculated based on a molecular size of 1.83 Mbp (*H. influenzae* RD KW20; GenBank accession number, NC000907). To ascertain the detection limit of the non-Hib LAMP assay, serial tenfold dilutions of genomic DNA were amplified and the results were compared with those of conventional PCR (Falla et al., 1994).

In the detection limit study, triplicate non-Hib LAMP testing was performed over a 3-day period using serial tenfold dilutions of genomic DNA. The supernatant of pooled *H. influenzae*-negative CSF specimens (Anh et al., 2006) was used in a spiking assay, and serial tenfold dilutions of genomic non-Hib DNA were amplified by the established LAMP assay.

### Non-Hib LAMP Primer Design

Five non-Hib LAMP primer sets were designed based on published sequences of the non-Hib capsulation loci (GenBank accession numbers, Z37516, HQ651151, Z33389, HM053635, and AF549211 for serotypes a, c, d, e, and f, respectively) using LAMP primer design software (FUJITSU LIMITED, 2016). The non-Hib LAMP primers included two outer primers (F3 and B3), a forward inner primer (FIP), a backward inner primer (BIP), and loop primers (LF and/or LB) (**Table 2A**).

**Table 2:** **(A)** LAMP Primer sequences in this study.

Serotyping primer name	LAMP primer sequence (sequence 5′-3′; reaction temperature, 63°C)	Length (base pairs)
**Serotype a**		
Hia_F3	ACT CAT TGC AGC ATT TGC	18
Hia_B3	AGA CAC AAT GAA TAT CTT CTG G	22
Hia_FIP	CGT GAA CAG GAA TAG TCC ACT CGA AAA TGC GGA TTA TAT TTA CGG	45
Hia_BIP	CCT ACA AGG AAC AAA GAC CAT CGG TGA CCG ATG TAT TAA TTT TGC C	46
Hia_LF	TTC TTT ATT AAA TTT TTT GAT GCC A	25
Hia_LB	AAC TAT TTT TAT CAA TGT CTC CTG G	25
**Serotype c**		
Hic_F3	TGG TTC AGT AGA TGA CTC AG	20
Hic_B3	CTG ATA TTT GTT TAT CGA CTT CAG	24
Hic_FIP	GGC TTG CCC ACC ATT TTC TTT ATC TAA GAT TAT TAA AAA ATG GCA GCG	48
Hic_BIP	TCT GCA AGA AAT GTT GGA ATT GAG CTT TTA CTA ACA AAA TCA TCA GGG TC	50
Hic_LF	AGA TAT ATG TGA TAT TTT TAG GAT	24
Hic_LB	ACG TTC AAA CCG AAT G	16
**Serotype d**		
Hid_F3	TCG ATA TTT CGT TAG AAC ATC TC	23
Hid_B3	CTA AGA AGA GTT TTA CAA CCA TTC	24
Hid_FIP	CTG AAA TGC AGA GGT TAA TTG CAT CCA ACT GCT TTT AAT TCA GAG CC	47
Hid_BIP	TCA AAG AAC TCT TTC TTC TTG GGA ATA AAC AGG TTG TAT CGG TCA TC	47
Hid_LB	GTA TGA TTA CCT TGT GAT TGA T	22
**Serotype e**		
Hie_F3	ATT GGA AAG GTC GCC GTA	18
Hie_B3	GTA ATA GCT GCC AGT GCT	18
Hie_FIP	CTC CAC TGC GAA AAG CTC AAC AAT GGA CAA GTC TAC CTC AA	41
Hie_BIP	GAG GGT TCT TTC AAA CTA TTG CTT GGC TTA GGG GTT TCT TCA CT	44
Hie_LB	GAC CAA CTT GTC TTA TCA ATC AAC A	25
**Serotype f**		
Hif_F3	TGA GTT ATA CAG TAT CGA TCT C	22
Hif_B3	TGT CAT CTG AAA AAT TTC TAA CGT	24
Hif_FIP	ACC CAA GAT AAG AAT TCT CTC TAA TTT ATA TCA ACT TGC TGT TCA A	46
Hif_BIP	TTG GAC TTG ATA GTA CCA AAA ACA GTT AGC AAC TAA ATT ACT ACC ATA	48
Hif_LF	CAT TCA TCA TTT TAA GTT GGC GTT	24
Hif_LB	GGC CTA TTT TTA TGA TAA ACA ACA C	25
		

### Non-Hib LAMP Reaction

The reaction mixture (25 μL) contained 1.6 μM each of FIP and BIP, 0.2 μM each of F3 and B3, 0.4 μM of LF, 8 U of *Bst* DNA polymerase large fragment (New England Biolabs, Ipswich, MA, United States), 1.4 mM deoxynucleoside triphosphates, 0.8 M betaine (Sigma, St. Louis, MO, United States), 20 mM Tris-HCl (pH 8.8), 10 mM KCl, 10 mM (NH_4_)_2_SO_4_, 8 mM MgSO_4_, 0.1% Tween 20, and template DNA (2 μL). Each serotype reaction mixture was incubated at 63°C for 60 min and then heated at 80°C for 2 min to terminate the reaction.

### Analysis of Non-Hib LAMP Products

A Loopamp real-time turbidimeter (LA-500; Eiken Chemical Co., Tokyo, Japan) was used to monitor the turbidity of the reaction tube in real-time by reading the optimal density at 650 nm (OD650) at 6-s intervals. The amplification time required to exceed a turbidity level of 0.1 (*Tt*) was calculated in accordance with the manufacturer’s protocol using the turbidimeter software (Mori et al., 2004).

For the detection limit study, a colorimetric visual inspection dye (leuco triphenylmethane (Miyamoto et al., 2015); D-QUICK, Kaneka Co., Osaka, Japan) and a thermostatic color sensor (MyAbscope^®^; Kaneka Co., Osaka, Japan; KANEKA, 2016) were used. The change in color of the reactions was observed in real time at 20-s intervals. The amplification time was determined when an absorbance level exceeded 0.1 using software of the thermostatic color sensor.

To verify their structure, the amplified LAMP products were sequenced by Akita Prefectural University Biotechnology Center using a BigDye^®^ Terminator V3.1 cycle sequencing kit (Applied Biosystems, Foster City, CA, United States) and a 3130xL Genetic Analyzer (Applied Biosystems). The primers used to sequence the target region are shown in Supplementary Table S1.

### Non-Hib PCR Assay

Non-Hib PCR using previously reported primers (Falla et al., 1994) was performed to type the capsule of *H. influenzae* strains (**Table 2B**). The PCR mixture (25 μL) consisted of 0.2 mM of each deoxyribonucleoside triphosphate, 10 mM Tris-HCl buffer (pH 8.3), 50 mM KCl, 2 mM MgCl_2_, 1 U Ex *Taq* DNA polymerase (Takara Bio, Tokyo, Japan), 0.5 μM of each primer, and 2 μL of template DNA. The PCR was performed using two thermal cyclers: Veriti^TM^ (Applied Biosystems, Foster City, CA, United States) in Japan and T-100^TM^ (Bio-Rad, Hercules, CA, United States) in South Korea. The reaction comprised 25 cycles, each involving 1 min of denaturation at 94°C, 1 min of annealing at 60°C, and 1 min of extension at 72°C. To detect serotype e, the reaction conditions were modified to use 35 cycles, each involving 30 s of denaturation at 94°C, 30 s of annealing at 45°C, and 30 s of extension at 72°C (Satola et al., 2007). Following the final cycle, all reactions were incubated for further 10 min at 72°C. Products were visualized by resolution in an agarose gel followed by staining with ethidium bromide.

**Table 2:** **(B)** PCR Primer sequences in this study (**Falla et al., 1994**).

Serotyping primer name	PCR primer sequence (sequence 5′-3′)	Length (base pairs)
**Serotype a**
a1	CTA CTC ATT GCA GCA TTT GC	20
a2	GAA TAT GAC CTG ATC TTC TG	20
**Serotype c**
c1	TCT GTG TAG ATG ATG GTT CA	20
c2	CAG AGG CAA GCT ATT AGT GA	20
**Serotype d**
d1	TGA TGA CCG ATA CAA CCT GT	20
d2	TCC ACT CTT CAA ACC ATT CT	20
**Serotype e**
e1	GGT AAC GAA TGT AGT GGT AG	20
e2	GCT TTA CTG TAT AAG TCT AG	20
**Serotype f**
f1	GCT ACT ATC AAG TCC AAA TC	20
f2	CGC AAT TAT GGA AGA AAG CT	20

### DNA Spiked Clinical CSF Specimens

To conduct a pilot evaluation of the non-Hib LAMP, seven *bexA* PCR-negative specimens were randomly selected from CSF collected during a 2-year prospective study of bacterial meningitis in Hanoi (Anh et al., 2006). CSF specimens were pre-treated at 95°C for 2 min and centrifuged (13,000 × g, 5 min). The supernatant of the seven clinical CSF specimens was preserved for use in DNA-spiked CSF experiments. A 2 μL aliquot of DNA-spiked CSF specimen was subjected to non-Hib serotyping PCR and non-Hib serotyping LAMP as described above at Hanyang University, South Korea.

### Ethics Statement

We utilized CSF specimens preserved from our previous surveillance study (Anh et al., 2006; Kennedy et al., 2007). All CSF specimens utilized in this study were de-identified prior to laboratory processing and analysis. Ethical approval for patient specimen collection during surveillance was obtained from the following ethics review committees: the Institutional Review Board of the International Vaccine Institute, Seoul, South Korea; and the Institutional Review Board at the National Institute of Hygiene and Epidemiology, Hanoi, Vietnam. Each institution participated in prospective, population-based surveillance for childhood meningitis from 1999 to 2002 (Anh et al., 2006; Kennedy et al., 2007). During those surveillance studies, written consent was not obtained as the CSF collection was considered routine standard care for hospitalized children with suspected bacterial meningitis. For this reason, verbal consent of the parent or legal guardian present with the child during the period of hospitalization was recorded in the patient’s medical chart at the time of the clinical lumbar puncture procedure. This consent procedure was approved by these local scientific ethics review committees of the participating institutions.

## Results

**FIGURE 1:**
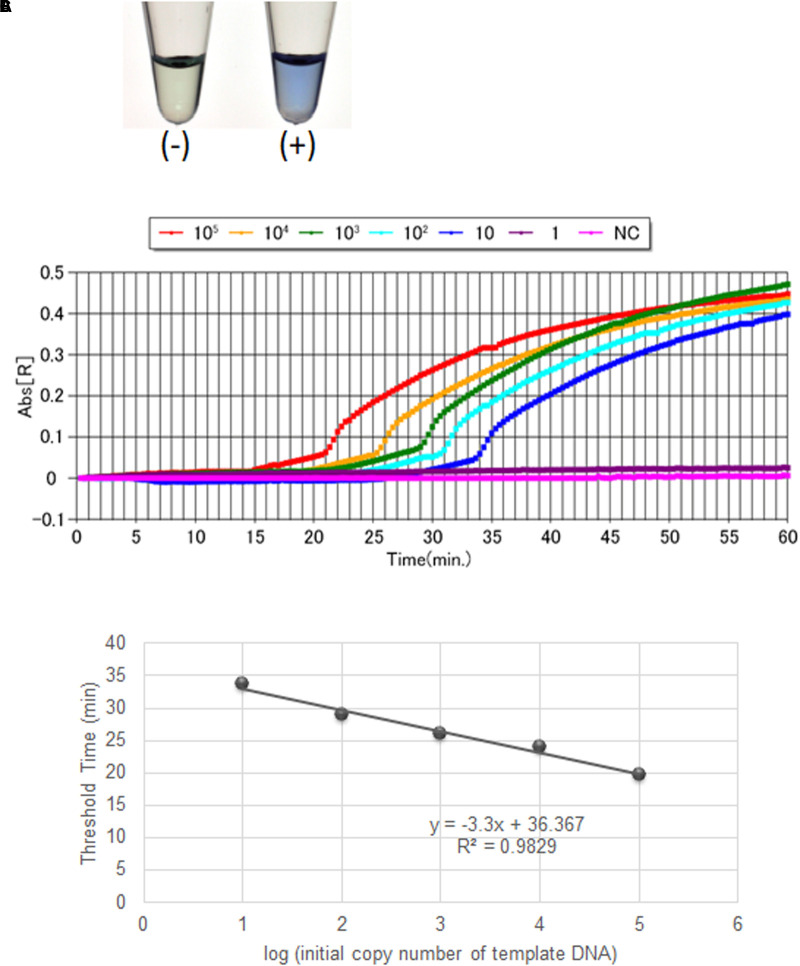
Visual inspection and dye-mediated monitoring of the *H. influenzae* serotype-specific LAMP assay. **(A)**, Detection of LAMP reaction using a colorimetric dye (leuco triphenylmethane; D-QUICK). The results were easily recognized by the naked eye, without UV-lamp. **(B)**, Results of a non-Hib LAMP assay (*H. influenzae* type a) using D-QUICK plus MyAbscope^®^. Numbers of bacteria decrease from left to right in the figure (100,000 to 1 copy number of DNA per reaction). **(C)**, Linear regression curve of LAMP results (*H. influenzae* type a) obtained using D-QUICK plus MyAbscope^®^.

The non-Hib LAMP assay for capsular types a, c, d, e, and f successfully amplified each target sequence of non-Hib capsulation loci (**Table 1**). The products were confirmed by visual inspection of turbidity/color of the reaction tube, real-time turbidimetry and real-time colorimetric sensor (**Figure 1**).

### Analytical Reactivity and Specificity of the Non-Hib LAMP

To evaluate the analytical reactivity and specificity of the non-Hib LAMP primers, we tested 42 *H. influenzae* strains and 8 non-*H. influenzae* strains. For each assay mixture, a standard genomic DNA concentration (10^5^ copies) was used for each strain. The non-Hib LAMP reaction for capsular types a, c, d, e, and f amplified each non-Hib target DNA sequence regardless of the different sources (i.e., respiratory tract or cerebrospinal fluid specimens). In contrast, genomic DNA of Hib or other target capsular types was not amplified. Neither non-typeable *H. influenzae* nor the eight standard non-*H. influenzae* species yielded a positive result in the non-Hib LAMP reaction (**Table 1**).

Amplification specificity was confirmed by sequencing, and the sequences were compared with those of the target region in the original sequence of the non-Hib capsulation locus (between F1 and B1; Supplementary Figure S1). The sequences obtained were identical to the expected nucleotide sequences (data not shown).

### Detection Limit of the Non-Hib LAMP Reaction

The detection limits of the LAMP assays for capsular types a, c, d, e, and f were 10^2^, 10^2^, 10^2^, 10^3^, and 10 copies per reaction, while those of the PCR assays were 10^4^, 10^4^, 10^3^, 10^3^, and 10^4^ genome copies per reaction, respectively. Thus, the sensitivity of the non-Hib LAMP assay was 1–1,000-fold greater than that of non-Hib PCR (**Table 3**). The detection limits of each serotype LAMP assay were identical between the real-time measurement and direct visual inspection. No amplification was apparent in the non-Hib LAMP reaction for samples lacking target DNA. The above results were obtained in triplicate over 3 days and identical results were obtained in the laboratories in Japan and South Korea.

**Table 3:** Detection limits of LAMP and PCR assays detecting DNAs of non-Hib serotypes and using the DNA spiked CSF specimens.

Non-Hib serotypes	Detection limit (Purified DNA)		Detection limit (Spiked CSF)	
	PCR^a,b^	LAMP^a^	PCR^a,b^	LAMP^a^
a	10^4^ copies^c^	10^2^	10^4^ copies^c^	10^2^
c	10^4^	10^2^	10^4^	10^2^
d	10^3^	10^2^	10^4^	10^2^
e	10^3^	10^3^	10^5^	10^3^
f	10^4^	10	10^4^	10

^a^*PCR results were obtained by electrophoretic analysis. LAMP results were determined visually. ^b^Conventional PCR (**Falla et al., 1994**). ^c^Amount of DNA per reaction*.

The results of LAMP assays using D-QUICK plus MyAbscope^®^ are shown in **Figure 1**. The color change of the LAMP products was more easily recognized than an increase in turbidity. The real-time measurement results were used to generate a linear regression curve with high linearity, while the observation period was longer than that of real-time turbidimetry (20-s vs. 6-s intervals) (**Figure 1C**). The total reaction time of the two methods was the same (60 min).

### LAMP Analysis of DNA-Spiked CSF Specimens

The detection limit of the non-Hib LAMP assay using DNA spiked CSF specimens was 10–1,000 genome copies, identical to that using purified DNA as the template (**Table 3**). However, that of the non-Hib PCR worsened from 10^3^ to 10^4^ genome copies per reaction for serotype d and from 10^3^ to 10^5^ genome copies per reaction for serotype e. The detection limit of the non-Hib LAMP assay was identical between direct visual inspection and real-time measurement.

## Discussion

This is the first report of a serotype-specific identification assay for *H. influenzae* using the LAMP method. The non-Hib LAMP assay established in this study accurately identified various standard and reference non-Hib strains. The non-Hib LAMP demonstrated an analytical specificity equivalent to that of non-Hib PCR. Notably, the non-Hib LAMP assay was found to have a detection limit 1–1,000-fold more sensitive than previously described non-Hib PCR methods (Falla et al., 1994; Corless et al., 2001). Although we used spiked CSF samples, the non-Hib LAMP reaction exhibited sensitivity equivalent to that when purified DNA was used as the template. However, the detection limit of the non-Hib PCR for serotypes d and e was reduced when spiked CSF samples were used. The superior detection limit and high robustness (Francois et al., 2011) of the LAMP assay may explain its superior detection limit to that of non-Hib PCR.

In children under 5 years of age, incidence of invasive non-Hib diseases has increased globally since the widespread introduction of Hib vaccines. Notably, in adults 65 years of age and older, the number of clinical infections with non-Hib strains have also increased recently (Desai et al., 2015). These studies suggest that the most common and important non-Hib strain are non-typeable, and encapsulated non-Hib strains (serotypes a, c, d, e, and f) began to be reported after the introduction of Hib vaccination (Dworkin et al., 2007; Resman et al., 2011; Ulanova and Tsang, 2014). Thus, the incidence of invasive non-Hib disease should be monitored and its clinical and epidemiological characteristics should be thoroughly investigated.

Existing assays for non-Hib infections have notable limitations. Conventional slide agglutination serotyping tests can produce misidentifications (LaClaire et al., 2003). However, in our study, the non-Hib LAMP assay successfully distinguished each non-Hib serotype strain. The non-Hib LAMP assay used in this study was analytically specific and had a superior detection limit to conventional PCR serotyping. Our previous reports of LAMP assays to detect *H. influenzae* type b, *S. pneumoniae* and *N. meningitidis* also revealed high sensitivity of the assays. The detection limits were 10-10^2^ DNA copies per reaction. The high sensitivity of the novel non-Hib LAMP assay is consistent with the previous studies (Kim et al., 2011, 2012; Lee et al., 2015a,b).

As the LAMP reaction progresses, the by-product pyrophosphate ions bind to magnesium ions and form a white precipitate of magnesium pyrophosphate. The resulting turbidity can be visualized by the naked eye. This characteristic feature of the LAMP reaction can be used to detect the reaction end-point, by identifying the presence of precipitate. The Loopamp real-time turbidimeter enables quantitative analysis of minute amounts of nucleic acids (Mori et al., 2004).

In the present study, we assessed the amount of non-Hib serotype template DNA in real-time using the D-QUICK plus MyAbscope^®^. To our knowledge, this is the first report of a LAMP assay using D-QUICK plus MyAbscope^®^ to quantify DNA. D-QUICK uses leuco triphenylmethane dye, which binds to double stranded DNA; a positive reaction is indicated by a change from colorless to violet (Miyamoto et al., 2015). This involves direct colorimetric detection of LAMP amplified products, rather than a by-product of the LAMP reaction, and the instrument measures the absorbance of the reaction in real time at 20-s intervals (KANEKA, 2016). As shown in **Figure 1C**, the real-time measurement had the linear regression curve with high linearity. Moreover, the simpler real-time absorbance measurement is less costly than the real-time turbidity measurement.

Previous studies demonstrated that the LAMP reaction is more tolerant of the presence of potentially perturbing biological substances (i.e., reaction inhibitors) than PCR (Kaneko et al., 2007). Therefore, LAMP assays may be suitable in resource-limited settings in developed and developing countries. The robust performance of the non-Hib LAMP assay (Francois et al., 2011) in the present study also suggests that LAMP-based detection of non-Hib and other invasive bacterial pathogens is feasible in a wide variety of clinical settings such as hospitals and health system clinical laboratories. In addition, compared with non-Hib PCR assays, the non-Hib LAMP assay has greater analytical sensitivity and less likely to miss clinical disease caused by non-Hib infections.

Our laboratory’s experience with the non-Hib LAMP and LAMP for the detection of other pathogens, such as *S. pneumoniae*, suggests that the cost of the non-Hib LAMP assay per specimen tested is markedly lower than that of PCR, and can be performed in laboratories with limited technology. Prospective studies of the non-Hib LAMP assay using clinical specimens that are now underway will confirm the assay’s sensitivity, specificity, predictive values, and likelihood ratios compared with bacterial culture, antigen detection, and PCR.

In summary, we have successfully established a LAMP-based non-Hib DNA amplification method and confirmed its superior analytical specificity and detection limit. Compared with PCR-based detection methods, this assay enables the detection of non-Hib serotypes with high sensitivity. Because the LAMP reaction is easy to set-up and does not require specialized equipment, it has obvious advantages in clinical settings and in population-based studies with limited access to well-equipped laboratories.

## Author Contributions

PK, DK, MS, and SH contributed the conception of this study; KT, DK, and MS designed the experiments; CT, KF, TI, DK, MS, and KT performed the experiments; PK acquired samples; CT, KT, DK, and MS analyzed data; CT, SH, PK, DK, and MS interpreted data; CT, PK, SH, DK, and MS drafted the manuscript; and KF, TI, and KT approved the manuscript.

## Conflict of Interest Statement

SH has received research grant funding from Kaneka Co., Ltd. KT is an employee of Kaneka Co., Ltd. The other authors declare that the research was conducted in the absence of any commercial or financial relationships that could be construed as a potential conflict of interest.

## Abbreviations

non-Hib: *Haemophilus influenzae* serotypes a, c, d, e and f.
