# Corrigendum: Molecular Serotype-Specific Identification of Non-type b *Haemophilus influenzae* by Loop-Mediated Isothermal Amplification

**DOI:** 10.3389/fmicb.2022.870651

**Published:** 2022-03-14

**Authors:** Chika Takano, Mitsuko Seki, Dong Wook Kim, Paul E. Kilgore, Kazumasa Fuwa, Koji Takahashi, Toshiaki Inazaki, Satoshi Hayakawa

**Affiliations:** ^1^Division of Microbiology, Department of Pathology and Microbiology, Nihon University School of Medicine, Tokyo, Japan; ^2^Department of Pharmacy, College of Pharmacy, Hanyang University, Ansan, South Korea; ^3^Institute of Pharmacological Research, Hanyang University, Ansan, South Korea; ^4^Department of Pharmacy Practice, Eugene Applebaum College of Pharmacy and Health Sciences, Wayne State University, Detroit, MI, United States; ^5^Medical Devices Solutions Vehicle, Kaneka Corporation, Hyogo, Japan; ^6^Nihon University School of Medicine, Tokyo, Japan

**Keywords:** loop-mediated isothermal amplification, *Haemophilus influenzae*, serotype identification, meningitis, cerebrospinal fluid

In the original article there was an error. In Table 2A, in the section Serotype c, Line 3, a nucleotide “A” should have been inserted at the 40th of Hic_FIP sequences and should read “Hic_FIP GGC TTG CCC ACC ATT TTC TTT ATC TAA GAT TAT TAA AAA ATG GCA GCG 48.”

**Table 2A:** LAMP Primer sequences in this study.

**Serotyping primer name**	**LAMP primer sequence** **(sequence 5^**′**^–3^**′**^; reaction temperature, 63**°**C)**	**Length (base pairs)**
**Serotype a**		
Hia_F3	ACT CAT TGC AGC ATT TGC	18
Hia_B3	AGA CAC AAT GAA TAT CTT CTG G	22
Hia_FIP	CGT GAA CAG GAA TAG TCC ACT CGA AAA TGC GGA TTA TAT TTA CGG	45
Hia_BIP	CCT ACA AGG AAC AAA GAC CAT CGG TGA CCG ATG TAT TAA TTT TGC C	46
Hia_LF	TTC TTT ATT AAA TTT TTT GAT GCC A	25
Hia_LB	AAC TAT TTT TAT CAA TGT CTC CTG G	25
**Serotype c**		
Hic_F3	TGG TTC AGT AGA TGA CTC AG	20
Hic_B3	CTG ATA TTT GTT TAT CGA CTT CAG	24
Hic_FIP	GGC TTG CCC ACC ATT TTC TTT ATC TAA GAT TAT TAA AAA ATG GCA GCG	48
Hic_BIP	TCT GCA AGA AAT GTT GGA ATT GAG CTT TTA CTA ACA AAA TCA TCA GGG TC	50
Hic_LF	AGA TAT ATG TGA TAT TTT TAG GAT	24
Hic_LB	ACG TTC AAA CCG AAT G	16
**Serotype d**		
Hid_F3	TCG ATA TTT CGT TAG AAC ATC TC	23
Hid_B3	CTA AGA AGA GTT TTA CAA CCA TTC	24
Hid_FIP	CTG AAA TGC AGA GGT TAA TTG CAT CCA ACT GCT TTT AAT TCA GAG CC	47
Hid_BIP	TCA AAG AAC TCT TTC TTC TTG GGA ATA AAC AGG TTG TAT CGG TCA TC	47
Hid_LB	GTA TGA TTA CCT TGT GAT TGA T	22
**Serotype e**		
Hie_F3	ATT GGA AAG GTC GCC GTA	18
Hie_B3	GTA ATA GCT GCC AGT GCT	18
Hie_FIP	CTC CAC TGC GAA AAG CTC AAC AAT GGA CAA GTC TAC CTC AA	41
Hie_BIP	GAG GGT TCT TTC AAA CTA TTG CTT GGC TTA GGG GTT TCT TCA CT	44
Hie_LB	GAC CAA CTT GTC TTA TCA ATC AAC A	25
**Serotype f**		
Hif_F3	TGA GTT ATA CAG TAT CGA TCT C	22
Hif_B3	TGT CAT CTG AAA AAT TTC TAA CGT	24
Hif_FIP	ACC CAA GAT AAG AAT TCT CTC TAA TTT ATA TCA ACT TGC TGT TCA A	46
Hif_BIP	TTG GAC TTG ATA GTA CCA AAA ACA GTT AGC AAC TAA ATT ACT ACC ATA	48
Hif_LF	CAT TCA TCA TTT TAA GTT GGC GTT	24
Hif_LB	GGC CTA TTT TTA TGA TAA ACA ACA C	25

In the original article there was an error. In Supplementary Figure 1B, an arrow of B2 should be from position 1840 to 1816; and in Supplementary Figure 1D, an arrow of LB should be from 3470 to 3494. Supplementary Figure 1 has been corrected and the **supplementary material** updated.

The authors apologize for this error and state that this does not change the scientific conclusions of the article in any way. The original article has been updated.

## Publisher's Note

All claims expressed in this article are solely those of the authors and do not necessarily represent those of their affiliated organizations, or those of the publisher, the editors and the reviewers. Any product that may be evaluated in this article, or claim that may be made by its manufacturer, is not guaranteed or endorsed by the publisher.

