# Supplementary material for: Molecular Serotype-Specific Identification of Non-type b Haemophilus influenzae by Loop-Mediated Isothermal Amplification
Source: Front Microbiol. 2017 Oct 4;8:1877. doi: 10.3389/fmicb.2017.01877 (PMC5632651; doi:10.3389/fmicb.2017.01877)
Supplement: Supplementary file 1 [file Table_1.DOCX]

**Table S1. F2 Primer sequences.**

| Primer name | PCR Primer Sequence (Sequence 5'-3') | Length  (base pairs) |
| --- | --- | --- |
| Hia_F2 | GAA AAT GCG GAT TAT ATT TAC GG | 23 |
| Hic_F2 | CTA AGA TTA TTA AAA AAT GGC AGC G | 25 |
| Hid_F2 | CAA CTG CTT TTA ATT CAG AGC C | 22 |
| Hie_F2 | CAA TGG ACA AGT CTA CCT CAA | 21 |
| Hif_F2 | TTA TAT CAA CTT GCT GTT CAA | 21 |
