# Supplementary material for: Molecular Serotype-Specific Identification of Non-type b Haemophilus influenzae by Loop-Mediated Isothermal Amplification
Source: Front Microbiol. 2017 Oct 4;8:1877. doi: 10.3389/fmicb.2017.01877 (PMC5632651; doi:10.3389/fmicb.2017.01877)
Supplement: Supplementary file 2 [file Image_1.PDF]

FIGURE S1. Nucleotide sequence of the *H. influenzae* capsulation locus genes used to design the non-Hib LAMP primers. The sequences used for the LAMP primers are indicated by arrows. (A) to (E) in the figure are the sequences of *H. influenzae* capsulation locus genes for serotypes a, c, d, e, and f, respectively.

Fig. S1(A)

```
3001                                                                    3070
TTGACGATGG TTCAAAAGAT AACACTGTTA ATAATGCAAA ATTAGCCGTT GCTAACGATA AAAGATTTAC

3071                                                                    3140
TATTCTTCGA AAAGAAAATT CTGGTGTTTC CTCTACTAGA AACTTTGGAA TAAAAAAAGG AAAAGGAGAA

3141                                                                    3210
TTCATTTTTT TCTGTGATTC TGATGACATT TTAGAAGGAA ATGCTATTGA TAAACTACTC ATTGCAGCAT
                                                             |--------F3-----

3211                                                                    3280
TTGCTGAAAA TGCGGATTAT ATTTACGGTG GCATCAAAAA ATTTAATAAA GAAAAAGAGT GGACTATTCC
---> |-----------F2----------><------------LF------------|   <---------F1----

3281                                                                    3350
TGTTCACGAC AAAAATAATC TATTCCTACA AGGAACAAAG ACCATCGATA AGAATACAGA ACTATTTTTA
-------|                  |-----------B1---------->             |------------

3351                                                                    3420
TCAATGTCTC CTGGGGCAAA ATTAATACAT CGGTCACTGT TAAAGAATAA ATTTTTTCCA GAAGATATTC
----LB--------><-----------B2----------|                      <-------B3-----

3421                                                                    3490
ATTGTGTCTA TTGTATCGGT AACTATATCT ACAAATATAC AGAAGATCAG GTCATATTCT TCAATATATT
--------|

3491                                                                    3560
TTTAGATGCT AAAAAAATCT ATTGTATCGG TAACTATATC TACAAATATA GAGAGCGAGA TTTAGAAAAC

3561                                   3600
AATGAACGAT CAATTACACA GCAACGTGAT ATAAAAGCCT
```

Fig. S1(B)

```
1619                                                                    1688
AATTATTAAA CTTTAAAAAT AATATATTTA TTATCTGTGT AGATGATGGT TCAGTAGATG ACTCAGCTAA
                                                  |---------F3--------->|---

1689                                                                    1758
GATTATTAAA AAATGGCAGC GTAAATATCC TAAAAATATC ACATATATCT ATAAAGAAAA TGGTGGGCAA
-----------F2---------->     <-----------LF-----------| <----------F1--------

1759                                                                    1828
GCCTCTGCAA GAAATGTTGG AATTGAGCAC GTTCAAACCG AATGGGTTAC TTTCATTGAC CCTGATGATT
--||-----------B1------------>|-------LB-------->             <-----B2------

1829                                                                    1898
TTGTTAGTAA AAACTATTTT TCTGAAGTCG ATAAACAAAT ATCAGAATCA GAGAATGTTT CACTAATAGC
-------------|         <-----------B3-----------|

1899          1912
TTGCCCTCTT GTTT
```

Fig. S1(C)

```
1                                                              60
GCAAATCATA GTGACAAAGA ACTGACTTTA CTAGACTACC AAGAAAGCTT AAAAACTGTA

61                                                            120
GATGTAGCTG TATTATTAGT TAACCATAAA GAATTTGTTG AAAATAAACC GCACTTTGGA

121                                                           180
AACAATGTTG TAATCGTTGA TACGAAAGGG ATTTGGTAAT TTTCACTATT ATAGGAAAAA

181                                                           240
ACTAAGTACT GGTAGAATTA ATTTATCTAC CAGTACTTTA TGTATATAAT TAGGTAAAAC

241                                                           300
AGATGAAGAT TTTAATTTTT GGTAGCTATG TTACAAGAGA TGCTTTTACT TTTGATAAAT

301                                                           360
CAAATGAATT TGAATTAGAT CGATATTTCG TTAGAACATC TCTAGCAACT GCTTTTAATT
                    |------------F3----------->    |---------F2-----

361                                                           420
CAGAGCCAAT AGAAGATAAT TATACGATGC AATTAACCTC TGCATTTCAG AAAAAAATTG
------>                    <------------F1------------|

421                                                           480
TAAATGCTGA GTTAAAAAAA GAATTCAAAG AACTCTTTCT TCTTGGGAAG TATGATTACC
                          |------------B1------------>|------LB---

481                                                           540
TTGTGATTGA TTTTATTGAT GACCGATACA ACCTGTTTAA ATTTAAGAAT GGTTGTAAAA
----------->      <-----------B2-----------|        <------B3------

541                                                           600
CTCTTCTTAG TGCTGAATTA AAAAATACTG ATTTCTTAGA TAAAAATCAA CATAACGGAG
---------|

601                                               645
AAATAATTTC TGGATTCTCT GAAGAATGGT TTGAAGAGTG GAAAA
```

Fig. S1(D)

```
3139                                                                    3208
AAAAGCTCTT AGTTATGAGC ACTCAATGTC ATTTCGATTG GGATACGCCC TCATTTTTGG TTTTAAATCA

3209                                                                    3278
TGGACAGGCT TTAAACATTT AATTAAAACA CTTTACACAC TACCTTTTGA GAAGAGAACT AAAAAACAAC

3279                                                                    3348
AACGTACAGA AATATCAGTT ACACTTCGCA AACCTGATTA TTTAACTATT AATTGGAAAG GTCGCCGTAC
                                                        |-------F3-------->

3349                                                                    3418
TATTACTCAA TGGACAAGTC TACCTCAAAA AGAGTTTAAG GATTTTATTG AGCTTTTCGC AGTGGAGCGG
       |---------F2---------->                     <---------F1---------|

3419                                                                    3488
CTTGAATTAA GAAACAGAGG GTTCTTTCAA ACTATTGCTT GCAAAAATAT TGACCAACTT GTCTTATCAA
                 |------------B1------------>           |--------LB---------

3489                                                                    3558
TCAACATTGA TAGTGAAGAA ACCCCTAAGC AGACTAAACA AGCACTGGCA GCTATTACAT TCTTAGATAA
-->         <--------B2--------|            <-------B3--------|

3559                                                                    3628
AGATAATAAT ACACTAGAAT CTAGTATTGA ATTACCACAA AGTAAAAAAC TGGGGAAATA CTATTTCTAC

3629                                                                    3698
CTTAATACAG AGAAAGATCA ACAGGATAAT TTATTTATTA TCCCACCTTT AGATTGTGAA AATATAAAAT

3699                                                                    3768
TAGATATTGT TCCTTGGGAT ATAAAAGGAA AAATATCTGT ACATAATAAA GTAGGCATAT CTCACTATAC

3769                                                                    3838
AAATGGTATT AGTATCATTC TTCCAACATA TAAAGGAGTT AATACTATTA AGAAATGCTT AGATTCTTTA
```

Fig. S1(E)

```
11861                                                                  11930
TTATTTTAAA GCCTGACGTT ATTATATACT CTCAACCTTC CTGTGATTTT TCTGCTTTAC TATATGAAAA

11931                                                                  12000
TGGATATTAT AACAATAGTA AATATTTTTC TAATATTATT CCATCAGTAG AAAGTGATGT TTTTAGATTC


12001                                                                  12070
AAAACTATTC AAGATAATTT AATTCAGATA CAGAATAATA TTATTATGCT ATCTAAACTA TGTGAGTTAT
                                                                     |--------

12071                                                                  12140
ACAGTATCGA TCTCTTTATA TCAACTTGCT GTTCAAATTC ATCTAAACGC CAACTTAAAA TGATGAATGA
-----F3-------> |---------F2---------->          <------------LF----------|

12141                                                                  12210
TATTATTAGA GAGAATTCTT ATCTTGGGTA TAAGGTTATT GATTTGGACT TGATAGTACC AAAAACAGAG
    <-----------F1-----------|                 |------------B1-----------> |-

12211                                                                  12280
GCCTATTTTT ATGATAAACA ACACTTAAAT GAATATGGTA GTAATTTAGT TGCTAATATT TATCTATATA
-----------LB------------>          <----------B2-----------|

12281                                                                  12350
ACGTTAGAAA TTTTTCAGAT GACATGCCAA AAAAAACAAT ACAAAAACAT CATATACAAA AAATAAATGG
<-----------B3-----------|

12351                                                                  12420
CGTCTTTGAA ATAACCTCAA ACAACTTAGA AAAAGAGAGC AACTCTATTT TGTTAAAAAT AAAAAACAAT

12421                                                                  12490
GAAAAACAAA ATCAAGATTT TGAAATAAAA ATAACATATT TCAATGAAGG AGAAATAATA AAGAATGATG

12491                                                                  12560
TAAAAAAAAT ATTATTGTTA CCATTGCATA GTATAGAATG CTCATATTTT ATAGAAGAAC TACATCAGAC

12561                                 12600
TAAAGTACTT ATAGAACCAA TTTCTATATC AAAAAATATT
```
